# Periodontitis and the subsequent risk of glaucoma: results from the real-world practice

**DOI:** 10.1038/s41598-020-74589-6

**Published:** 2020-10-16

**Authors:** Kuo-Ting Sun, Te-Chun Shen, Shih-Chueh Chen, Chia-Ling Chang, Ching‐Hao Li, Xin Li, Kalaiselvi Palanisamy, Ning-Yi Hsia, Wen-Shin Chang, Chia-Wen Tsai, Da-Tian Bau, Chi-Yuan Li

**Affiliations:** 1 School of Dentistry, China Medical University, Taichung, Taiwan; 2 Department of Pediatric Dentistry, China Medical University Hospital, Taichung, Taiwan; 3 Department of Internal Medicine, China Medical University Hospital, Taichung, Taiwan; 4 School of Medicine, China Medical University, Taichung, Taiwan; 5 Department of Endocrinology, Cheng Ching Hospital, Taichung, Taiwan; 6 Management Office for Health Data, China Medical University Hospital, Taichung, Taiwan; 7 Graduate Institute of Biomedical Sciences, China Medical University, Taichung, Taiwan; 8 Department of Ophthalmology, China Medical University Hospital, No. 2 Yude Road, Taichung, 404 Taiwan; 9 Terry Fox Cancer Research Laboratory, Department of Medical Research, China Medical University Hospital, Taichung, Taiwan

**Keywords:** Diseases, Health care, Risk factors

## Abstract

Periodontitis is a multifactorial inflammatory disease that can cause tooth loss and contribute to systemic inflammation. It is suggested that periodontitis may be associated with the development of glaucoma. Based on data from Taiwan’s National Health Insurance Research Database, a retrospective cohort study was conducted to investigate the risk of developing glaucoma in patients with periodontitis. The periodontitis cohort consisted of newly diagnosed adult patients (*n* = 194,090, minimum age = 20 years) between 2000 and 2012. The comparison group included age-, gender-, and diagnosis date-matched people without periodontitis (*n* = 194,090, minimum age = 20 years). Incident glaucoma was monitored until the end of 2013. Hazard ratios (HRs) with confidence intervals (CIs) were established based on the Cox proportional hazard models. The risk of developing glaucoma was higher in patients with periodontitis than those without periodontitis (31.2 vs. 23.3 patients per 10,000 person-years, with an adjusted HR of 1.26 [95% CI 1.21–1.32]). A high risk was evident even after stratifying by age (adjusted HRs = 1.34 [1.26–1.44] for ages 20–49, 1.24 [1.13–1.36] for ages ≥ 65, and 1.20 [1.12–1.29] for ages 50–64 years), sex (adjusted HRs = 1.33 [1.24–1.41] and 1.21 [1.14–1.28] for men and women, respectively), presence of comorbidity (adjusted HRs = 1.38 [1.29–1.47] and 1.18 [1.12–1.25] for without and with comorbidity, respectively), and corticosteroid use (adjusted HRs = 1.27 [1.21–1.33] and 1.21 [1.08–1.35] for without and with corticosteroid use, respectively). Specifically, patients with periodontitis exhibited a significantly high risk of primary open-angle glaucoma (adjusted HR = 1.31 [1.21–1.32]) but not for primary closed-angle glaucoma (adjusted HR = 1.05 [0.94–1.17]). People with periodontitis are at a greater risk of glaucoma than individuals without periodontitis. Ocular health should be emphasized for such patients, and the underlying mechanisms need further investigation.

## Introduction

Periodontitis is a common disease worldwide that features inflammation of the gums and supporting tooth structures^1,2^. Periodontal health is important for maintaining an adequate quality of life, and poor periodontal conditions can lead to pain, tooth loss, and malnutrition^3^. In addition, periodontal plaque can induce local and even systemic inflammation^4^. Association was reported between periodontitis and various systemic diseases, including atherosclerosis^5^, diabetes mellitus^6^, metabolic syndrome^7^, osteoporosis^8^, rheumatoid arthritis^9^, and respiratory diseases^10^. In addition, periodontitis can affect the development of ocular diseases^11,12^.

Belonging to a group of progressive optic neuropathies, glaucoma represents a disease featuring degenerative changes in both retinal ganglion cells and optic nerve, mostly due to high intraocular pressure (IOP)^13^. Primary open-angle glaucoma (POAG) is the most common glaucoma type, accounting for approximately 80% of all cases, followed by primary closed-angle glaucoma (PCAG)^14^. The results of population-based studies suggest that glaucoma is the leading cause of blindness globally, with an estimated 60 million people worldwide having visual impairment due to glaucoma^15,16^. Although its etiology is still not sufficiently understood, there are numerous risk factors, from genetic to environmental factors, such as family history, race, age, high IOP^13^, lifestyle, sleep quality, diet, exercise^17^, corticosteroid use^18^, and inflammation^19,20^.

There has been limited interest in the relationship between periodontitis and glaucoma. In fact, periodontitis increased the systemic inflammatory reaction, and glaucoma, as a neurodegenerative disease, could be exacerbated by the result of the chronic systemic inflammation^4,19^. In a case–control study including 119 POAG cases and 78 controls, Polla et al.^21^ reported that patients with POAG have fewer natural teeth and higher number of oral bacteria (Streptococci) than those without POAG. Pasquale et al.^22^ conducted a prospective study (40,536 men) showing a lack of association between POAG and tooth number, periodontal disease, or root canal treatment. However, they reported that within the past 2 years, both losing teeth and having a prevalent periodontal disease diagnosis were associated with a 1.85-fold increased risk of POAG. The results of previous studies were inconsistent and showed some limitations, such as relatively low sample size^21^ and inclusion of only men^22^. Therefore, we conducted a retrospective population-based cohort study based on Taiwan’s National Health Insurance Research Database (NHIRD) to clarify the potential association of periodontitis and development of glaucoma and its subtypes, POAG and PCAG.

## Materials and methods

### Data source

The National Health Insurance (NHI) program was established in 1995 in Taiwan and contains information on more than 99.9% of residents to date. The NHIRD is managed and updated by the National Health Research Institutes. For this study, we selected a subset of NHIRD marked as the Longitudinal Health Insurance Database 2000 (LHID2000). This database includes medical claim information of 1,000,000 people randomly selected in 2000 and data on demographic status, diagnostic codes, and medication and procedure claims between 1995 and 2013. This study was conducted with permission from the ethics committee (Research Ethics Committee of the China Medical University and Hospital [CMUH-104-REC2-115]). All methods were performed following the Strengthening the Reporting of Observational Studies in Epidemiology guideline. Informed consent was unnecessary for the de-identified data and waived by the Research Ethics Committee of the China Medical University and Hospital.

### Study population

**Figure 1:**
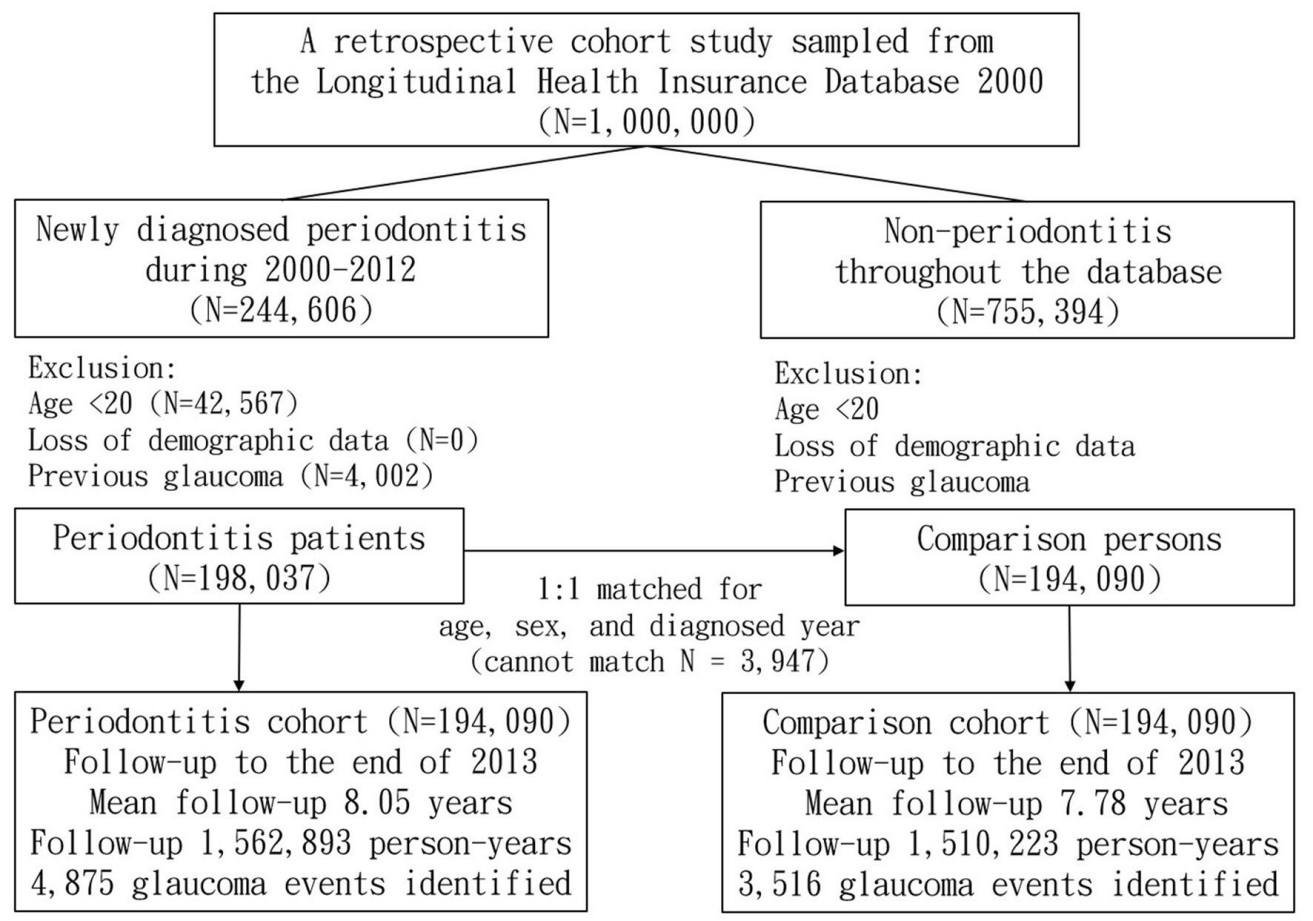
Flow chart showing subject selection, follow-up time, and identified events.

Adult patients in whom periodontitis (International Classification of Diseases, 9th Revision, Clinical Modification [ICD-9-CM] codes 523.3 and 523.4) was first diagnosed (index date) between 2000 and 2012 were chosen for the periodontitis cohort (*n* = 194,090, minimum age = 20 years). We excluded those with age < 20 years, incomplete age and sex information, as well as those diagnosed with glaucoma before the index date. The comparison group included people without periodontitis, and these individuals were age-, gender-, and index year-matched with the periodontitis cohort (*n* = 194,090, minimum age = 20 years). This group also included only individuals with complete information, similar to the periodontitis cohort. All participants (*n* = 388,180) were monitored until the first record of any of the following: development of glaucoma, withdrawal from the NHI program, death, or the end of 2013 (Fig. 1).

### Study outcome and comorbidities

The occurrence of glaucoma (ICD-9-CM code 365) was the primary outcome. We further identified two documented subtypes of glaucoma: POAG (with ICD-9-CM code 365.1) and PCAG (with ICD-9-CM code 365.2)^23^. In addition, we collected several comorbidities related to glaucoma and the most related medication, corticosteroid, as potential confounders. Detailed comorbidities assessed included the presence of cardiometabolic diseases, such as hypertension (ICD-9-CM codes 401–405), diabetes mellitus (ICD-9-CM code 250) and hyperlipidemia (ICD-9-CM code 272), migraine (ICD-9-CM code 346), asthma/chronic obstructive pulmonary disease (COPD) (ICD-9-CM codes 493 and 496), chronic liver disease and cirrhosis (CLD; ICD-9-CM code 571), chronic kidney disease (CKD; ICD-9-CM code 585), and rheumatic diseases (ICD-9-CM codes 446.5, 710.0–710.4, 714.0–714.2, 714.8, and 725).

### Statistical analysis

Chi-square test and *t*-test were used to compare the distribution of baseline characteristics between the groups (periodontitis vs. nonperiodontitis) for categorical and continuous variables, respectively. To evaluate the cumulative incidence of glaucoma in both groups, we created the Kaplan–Meier curves followed by testing inter-group differences with a log-rank test. Cox proportional hazard models were used to estimate the hazard ratios (HRs) along with 95% confidence intervals (CIs). The multivariate Cox model was applied to estimate the adjusted HRs (aHRs) after controlling for age, sex, comorbidities, and corticosteroid use, which were significant in the univariate model. For further data analysis, we assessed the effects of periodontitis on the risk of POAG and PCAG. Similar analyses were performed using univariate and multivariate Cox proportional hazard models. All the analyses were performed using STATA statistical software (StataCorp. 2015, R 14, StataCorp LP). Statistical significance was determined using a two-tailed test, and *p*-values were considered significant if lower than 0.05.

## Results

We recruited the periodontitis and comparison cohorts consisting of 194,090 adult persons each (Table 1). The distributions of age and gender were similar between the periodontitis and comparison groups. The periodontitis cohort had an average age of 42.5 ± 15.1 years. In both cohorts, 51.0% of the individuals were women. Compared with individuals without periodontitis, patients with periodontitis had a significantly higher prevalence of hypertension, CLD, hyperlipidemia, diabetes mellitus, asthma/COPD, migraine, rheumatic diseases, and corticosteroid use (*p* < 0.05).

**Table 1:** Baseline characteristics of the periodontitis cohort and control group of patients.

	Periodontitis				*p*-value^#^
	No		Yes		
	N = 194,090		N = 194,090		
	n	%	n	%	
**Age (years)**					Matching factor
20–49	135,730	69.9	135,730	69.9	
50–64	40,420	20.8	40,420	20.8	
≥ 65	17,940	9.24	17,940	9.24	
Mean ± SD	42.5	± 15.1	42.5	± 15.1	
**Gender**					Matching factor
Women	98,942	51.0	98,942	51.0	
Men	95,148	49.0	95,148	49.0	
**Comorbidity**					
Hypertension	30,742	15.8	33,608	17.3	< 0.0001
Diabetes mellitus	14,512	7.48	16,817	8.66	< 0.0001
Hyperlipidemia	20,977	10.8	27,229	14.0	< 0.0001
Migraine	5048	2.60	6262	3.23	< 0.0001
Asthma/COPD	13,027	6.71	14,979	7.72	< 0.0001
CLD	23,616	12.2	30,246	15.6	< 0.0001
CKD	1747	0.90	1798	0.93	0.39
Rheumatic diseases	3487	1.80	4749	2.45	< 0.0001
**Medication**					
Corticosteroid use	16,661	8.58	20,761	10.7	< 0.0001

*COPD* chronic obstructive pulmonary disease, *CLD* chronic liver disease and cirrhosis, *CKD* chronic kidney disease, *SD* standard deviation.

^#^Chi-squired test and t-test.

As shown in Table 2, the overall incidence rates of glaucoma in the periodontitis and comparison groups were 31.2 and 23.3 (per 10,000 person-years), respectively. Compared with that in the comparison group, the aHR for glaucoma in the periodontitis cohort was 1.26 (95% CI 1.21–1.32) after controlling for the effects of age, sex, comorbidities, and corticosteroid use. Higher age was associated with a higher risk of glaucoma, with aHRs of 2.65 (95% CI 2.51–2.80) and 3.43 (95% CI 3.21–3.67) for patients aged 50–64 and over 65 years, respectively, compared with those aged between 20 and 49 years. The patients with diabetes showed a higher risk of glaucoma (aHR = 1.60, 95% CI 1.50–1.70) than those without diabetes. The risk of glaucoma was also increased in patients with hyperlipidemia (aHR = 1.27, 95% CI 1.20–1.35), hypertension (aHR = 1.26, 95% CI 1.19–1.33), corticosteroid use (aHR = 1.26, 95% CI 1.18–1.35), CKD (aHR = 1.19, 95% CI 1.02–1.39), rheumatic diseases (aHR = 1.17, 95% CI 1.04–1.31), and CLD (aHR = 1.12, 95% CI 1.06–1.19) than in individuals without these comorbidities or medication.

**Table 2:** Analysis of risk factors for development of glaucoma.

	Event	PY	Rate^#^	Crude HR (95% CI)	Adjusted HR^†^ (95% CI)
**Periodontitis**					
No	3516	1,510,223	23.3	Reference	Reference
Yes	4875	1,562,893	31.2	1.34 (1.28–1.40)***	1.26 (1.21–1.32)***
**Age**					
20–49	3426	2,229,939	15.4	Reference	Reference
50–64	3082	600,847	51.3	3.37 (3.21–3.54)***	2.65 (2.51–2.80)***
≥ 65	1883	242,330	77.7	5.14 (4.86–5.44)***	3.43 (3.21–3.67)***
**Gender**					
Women	4403	1,574,697	28.0	Reference	Reference
Men	3988	1,498,419	26.6	0.95 (0.91–0.99)*	0.98 (0.93–1.02)
**Comorbidity**					
Hypertension					
No	5532	2,632,045	21.0	Reference	Reference
Yes	2859	441,071	64.8	3.13 (2.99–3.28)***	1.26 (1.19–1.33)***
Diabetes mellitus					
No	6658	2,862,724	23.3	Reference	Reference
Yes	1733	210,392	82.4	3.59 (3.41–3.79)***	1.60 (1.50–1.70)***
Hyperlipidemia					
No	6284	2,749,410	22.9	Reference	Reference
Yes	2107	323,706	65.1	2.90 (2.76–3.05)***	1.27 (1.20–1.35)***
Migraine					
No	8096	2,996,731	27.0	Reference	Reference
Yes	295	76,385	38.6	1.45 (1.29–1.62)***	1.06 (0.94–1.19)
Asthma/COPD					
No	7446	2,885,909	25.8	Reference	Reference
Yes	945	187,207	50.5	1.98 (1.85–2.12)***	1.00 (0.93–1.08)
CLD					
No	6658	2,689,128	24.8	Reference	Reference
Yes	1733	383,988	45.1	1.84 (1.74–1.94)***	1.12 (1.06–1.19)***
CKD					
No	8226	3,052,603	27.0	Reference	Reference
Yes	165	20,513	80.4	3.03 (2.60–3.54)***	1.19 (1.02–1.39) *
Rheumatic diseases					
No	8084	3,018,144	26.8	Reference	Reference
Yes	307	54,972	55.9	2.11 (1.88–2.36)***	1.17 (1.04–1.31)***
**Medication**					
Corticosteroid use					
No	7106	2,847,211	25.0	Reference	Reference
Yes	1285	225,905	56.9	2.34 (2.20–2.48)***	1.26 (1.18–1.35)***

*CI* confidence interval, *CKD* chronic kidney disease, *CLD* chronic liver disease and cirrhosis, *COPD* chronic obstructive pulmonary disease, *HR* hazard ratio, *PY* person-years.

^#^Incidence rate per 10,000 person-years;

^†^Multivariable analysis including age, gender, comorbidities, and corticosteroid use;

*p < 0.05, ***p < 0.001.

Table 3 displays the relationship between glaucoma and periodontitis after stratifying patients by age, gender, comorbidity, and corticosteroid use. The aHRs for glaucoma were high in all three age groups of individuals in the periodontitis cohort compared with that in the comparison group. The aHRs for glaucoma were 1.21 and 1.33 in women and men, respectively, in the periodontitis cohort compared with that in the comparison group. The aHRs for glaucoma were 1.38 (95% CI 1.29–1.47) and 1.18 (95% CI 1.12–1.25) in individuals without and with any comorbidity in the periodontitis cohort compared with that in the comparison group, respectively. Lastly, the aHRs for glaucoma were 1.27 (95% CI 1.21–1.33) and 1.21 (95% CI 1.08–1.35) in individuals without and with corticosteroid use in the periodontitis cohort compared with that in the comparison group, respectively.

**Table 3:** Incidences and hazard ratios of glaucoma for individuals with and without periodontitis stratified by age, gender, comorbidity, and corticosteroid use.

	Periodontitis						Crude HR (95% CI)	Adjusted HR^†^ (95% CI)
	No			Yes				
	Event	PY	Rate^#^	Event	PY	Rate^#^		
**Age**								
20–49	1400	1,100,619	12.7	2026	1,129,321	17.9	1.41 (1.31–1.51)***	1.34 (1.26–1.44)***
50–64	1336	295,198	45.3	1746	305,648	57.1	1.26 (1.17–1.35)***	1.20 (1.12–1.29)***
≥ 65	780	114,406	68.2	1103	127,925	86.2	1.27 (1.16–1.40)***	1.24 (1.13–1.36)***
**Gender**								
Women	1920	776,981	24.7	2483	797,717	31.1	1.26 (1.19–1.34)***	1.21 (1.14–1.28)***
Men	1596	733,242	21.8	2392	765,177	31.3	1.43 (1.35–1.53)***	1.33 (1.24–1.41)***
**Comorbidity**^**‡**^								
No	1584	1,082,235	14.6	2021	1,026,924	19.7	1.34 (1.25–1.43)***	1.38 (1.29–1.47)***
Yes	1932	427,988	45.1	2854	535,970	53.3	1.18 (1.11–1.25)***	1.18 (1.12–1.25)***
**Corticosteroid**								
No	3033	1,414,281	21.5	4073	1,432,931	28.4	1.32 (1.26–1.39)***	1.27 (1.21–1.33)***
Yes	483	95,942	50.3	802	129,963	61.7	1.22 (1.09–1.37)***	1.21 (1.08–1.35)**

*PY* person-years, *HR* hazard ratio, *CI* confidence interval.

^#^Incidence rate per 10,000 person-years.

^†^Multivariable analysis including age, gender, comorbidities, and corticosteroid use.

^‡^Comorbidity group contains individuals with any comorbidity of the following (hypertension, diabetes mellitus, hyperlipidemia, migraine, asthma/COPD, CLD, CKD and rheumatic disease).

** *p* < 0.01, ***p < 0.001.

**Figure 2:**
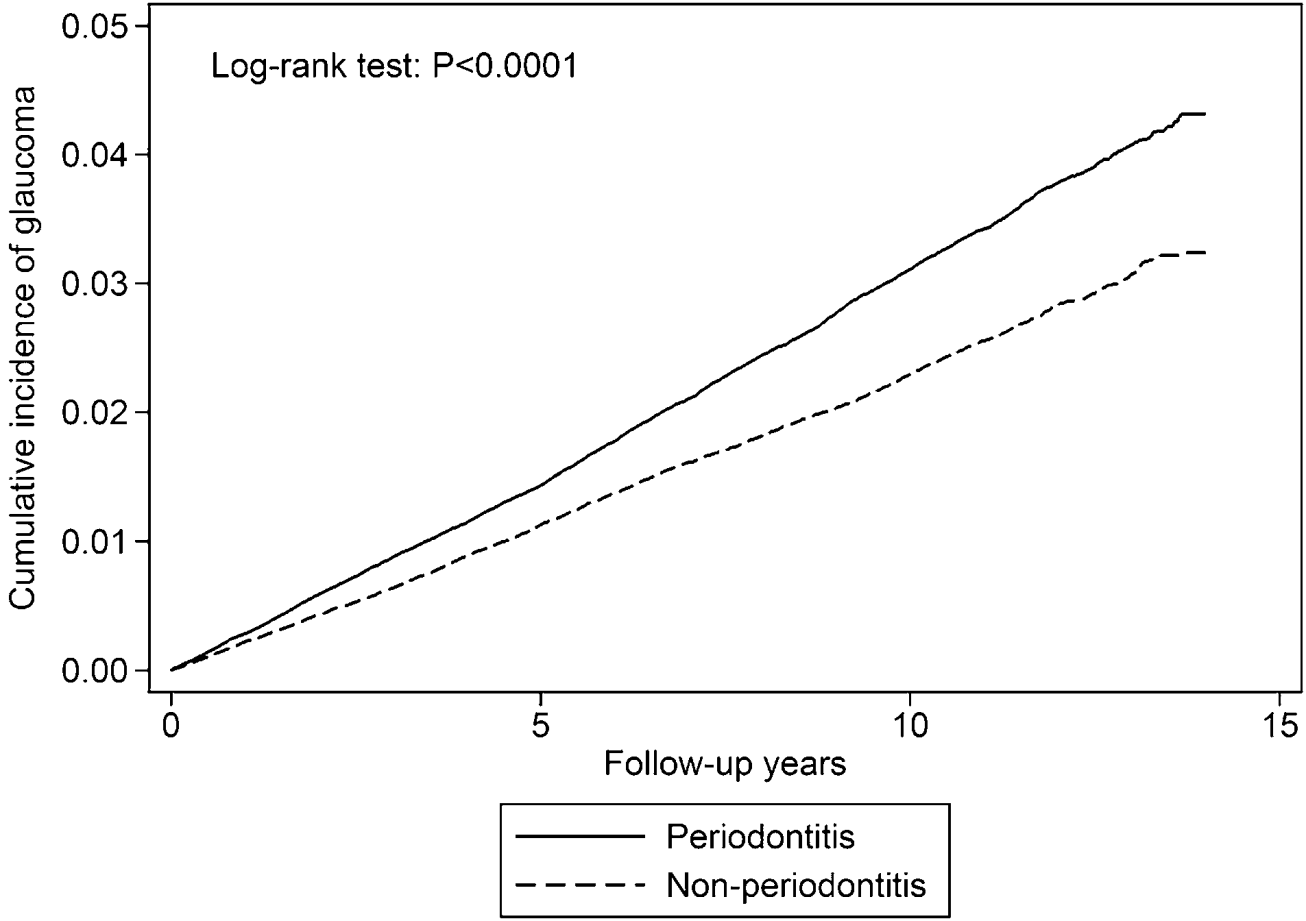
Cumulative incidence of glaucoma in the periodontitis and comparison groups.

We further identified the documented POAG (ICD-9-CM 365.1, *n* = 1219) and PCAG (ICD-9 CM 365.2, *n* = 1264) cases in overall glaucoma cases (*n* = 8391; Table 4). Compared with individuals without periodontitis, patients with periodontitis had a significant relationship with POAG (aHR = 1.31, 95% CI 1.17–1.47) but not with PCAG (aHR = 1.05, 95% CI 0.94–1.17). Cumulative incidences of glaucoma in individuals with and without periodontitis are illustrated in Fig. 2. The log-rank test showed that the patients with periodontitis demonstrated a significantly increased cumulative incidence of glaucoma compared with the comparison group (*p* < 0.0001).

**Table 4:** Incidences and hazard ratios of documented POAG and PCAG for individuals with and without periodontitis.

	Periodontitis	
	No	Yes
**Overall glaucoma**	**n = 8391**	
Event	3516	4875
Rate^#^	23.3	31.2
Crude HR (95% CI)	Reference	1.34 (1.28–1.40)***
Adjusted HR (95% CI)^†^	Reference	1.26 (1.21–1.32)***
**Confirmed POAG (ICD-9-CM 365.1)**	**n = 1219**	
Event	499	720
Rate^#^	3.30	4.61
Crude HR (95% CI)	Reference	1.39 (1.24–1.56)***
Adjusted HR (95% CI)^†^	Reference	1.31 (1.17–1.47)***
**Confirmed PCAG (ICD-9-CM 365.2)**	**n = 1264**	
Event	587	677
Rate^#^	3.89	4.33
Crude HR (95% CI)	Reference	1.11 (1.00–1.24)
Adjusted HR (95% CI)^†^	Reference	1.05 (0.94–1.17)

*HR* hazard ratio, *CI* confidence interval, *ICD-9-CM* international classification of diseases, 9th revision, clinical modification, *PCAG* primary closed-angle glaucoma, *POAG* primary open-angle glaucoma.

^#^Incidence rate per 10,000 person-years.

^†^Multivariable analysis including age, gender, comorbidities, and corticosteroid use.

***p < 0.001.

## Discussion

This retrospective population-based cohort study analyzed the occurrence of glaucoma in individuals with periodontitis and in a comparison group of individuals without periodontitis. Results showed that compared with people without periodontitis, the presence of periodontitis is associated with a significant risk of glaucoma, although the reduction is in the magnitude of the risk after adjustment. As expected, the risk of glaucoma increased in older people, those with comorbidity, and those with corticosteroid use. Furthermore, glaucoma was more likely in the periodontitis group than in the comparison group even after stratification by age, sex, presence of comorbidity, or corticosteroid use. Moreover, patients with periodontitis had more association with POAG than PCAG.

It is important to note that the incidence of glaucoma increased with age in both the periodontitis and comparison cohorts. However, the crude and adjusted periodontitis to nonperiodontitis HRs were higher in the youngest age group. Similarly, the incidence of glaucoma increased with the presence of comorbidity and with corticosteroid use in both the periodontitis and comparison cohorts. However, the crude and adjusted periodontitis to nonperiodontitis HRs were higher in the noncomorbidity and noncorticosteroid use groups (Table 3). This phenomenon reflects that periodontitis alone is associated with glaucoma risk. However, age, comorbidity, and corticosteroid use could further modify the relationship (having more impact on the nonperiodontitis group than the periodontitis group).

The mechanisms between periodontitis and glaucoma remain uncertain, but several hypotheses have been suggested. First, oral microbiome from periodontitis can cause immune responses and exacerbate glaucomatous neurodegeneration. Astafurov et al.^24^ reported that patients with glaucoma had higher bacterial loads in the oral cavity compared with people without glaucoma. In an animal model, they also found that the administration of lipopolysaccharide in mice could enhance the development of glaucoma via the upregulation of the complement system and toll-like receptor 4-signaling activity along with microglial activation in the optic nerve^24^. Second, endothelial cell dysfunction can be involved in the pathophysiology of glaucoma^25^. Periodontitis could induce chronic subclinical systemic inflammation leading to endothelial cell dysfunction. Endothelial dysfunction can lead to impaired flow-mediated vasodilation that causes poor perfusion of the optic nerve, which contributes to glaucoma development^26^. In addition, bacterial products can directly be linked to neurodegeneration. Neurotoxicity from some pathogenic species could be mediated by nitric oxide production through effects on microglia and astrocytes^27^. The localization of bacteria in these structures may not be necessary, and the bacterial products can initiate a local inflammatory response that leads to glaucomatous neurodegeneration^21^.

PCAG is less common than POAG; however, it is more prevalent in Asian countries^28^. PCAG is more common in older people and women, as well as in individuals with shallow anterior chamber or short axial length (hypermetropic eye) that is based on the pupillary block together with the anterior movement of the lens^29^. In addition, Chen and Lin^23^ reported that patients with PCAG are associated with comorbid cataracts and certain systemic or distant diseases (headaches, peptic ulcer, hyperlipidemia, and liver diseases). However, compared with POAG, PCAG is less associated with systemic diseases^29^. Our findings are in accordance with the above concept, that is, we found that patients with periodontitis had more association with POAG than PCAG. Incident PCAG was higher in the periodontitis group than in the comparison group, but the statistical significance was not reached in our analysis. Because the confirmed subtype (PAOG and PCOG) was only 30% of the total glaucoma events in the study, the real number of PAOG and PCOG was much underestimated. Therefore, the precise association between periodontitis and glaucoma subtype needs further investigation.

The present study’s strength primarily stems from population-based data to enroll sufficient periodontitis (*n* = 194,040) and nonperiodontitis (*n* = 194,040) cases to evaluate glaucoma development. Taiwan’s NHIRD is a large database with nationwide coverage, and no difference was found in the demographic distribution between LHID2000 and the original NHIRD. In addition, the universal coverage (> 99.9%) in the insurance program ensures that all citizens can have no access barriers to health care, irrespective of socioeconomic factors^30^. The NHIRD allowed us to reflect a “real-world” scenario in which periodontitis, glaucoma, and other comorbidities were diagnosed directly during medical consultation^31,32^.

However, our study had some limitations. First, diagnoses were based on ICD format (for periodontitis, glaucoma, and comorbidities), which is strongly dependent on the performance of physicians. The definition of cases and events may be inconsistent. Audits were regularly performed to ensure that negligence and misdiagnoses were kept to a minimum. Another limitation is that the NHI research center did not collect all comprehensive data that may be confounding factors (e.g., smoking and alcohol consumption habits, physical activity and diet style, occupation, body mass index, family history, and environmental exposure). Moreover, the database did not contain some important clinical variables (e.g., dental and ocular findings, laboratory data such as inflammatory markers, culture results, and pathologic reports). Therefore, the severity of the disease or the disease subtypes for periodontitis and glaucoma could not be precisely evaluated. Finally, one must bear in mind that the study could be biased because of possible unmeasured or unknown confounding variables.

## Conclusion

Patients with periodontitis may increase the risk of glaucoma development compared with individuals without periodontitis. The association between periodontitis and glaucoma remained statistically significant regardless of age, gender, presence of comorbidity, and corticosteroid use. Particularly, patients with periodontitis exhibited a higher risk of POAG. Ocular health should be emphasized for such patients, and the underlying mechanisms need further investigation.

## Data Availability

The datasets analyzed in the current study can be accessed from the Taiwan National Health Insurance Research Database repository (https://nhird.nhri.org.tw/en).
